# The Prevalence and Impact of Dentinal Hypersensitivity on Adults’ Quality of Life in Saudi Arabia

**DOI:** 10.3390/dj13080353

**Published:** 2025-08-04

**Authors:** Haya Alayadi, Omar Alsadon, Maram Ali Alwadi, Alaa A. Alkhateeb, Deema Alroweilly, Zainab Alassmi, Wedad Alshehri

**Affiliations:** Dental Health Department, College of Applied Medical Sciences, King Saud University, Riyadh 11451, Saudi Arabia; oalsadon@ksu.edu.sa (O.A.); malwadi@ksu.edu.sa (M.A.A.); alkhateeb@ksu.edu.sa (A.A.A.); dalroweilly@ksu.edu.sa (D.A.); zalasmi@ksu.edu.sa (Z.A.); walshuhri@ksu.edu.sa (W.A.)

**Keywords:** dentin sensitivity, quality of life, oral health, public health

## Abstract

**Background:** Dentinal hypersensitivity (DH) significantly impacts oral health-related quality of life. While global prevalence estimates range from 10–15%, region-specific data from Saudi Arabia remain limited. This study also aligns with Saudi Vision 2030’s mental health initiatives, as DH-associated anxiety impacts overall well-being. This study assessed DH prevalence and quality of life impact among Saudi adults. **Methods:** A cross-sectional online survey was conducted among 748 Saudi adults aged ≥ 18 years between April and May. Data were collected using a validated Arabic Dentinal Hypersensitivity Experience Questionnaire (DHEQ) alongside socio-demographic variables. Participants reporting DH symptoms within 12 months were included in impact analyses. Descriptive statistics and one-way ANOVA examined associations between DHEQ scores and participant characteristics. **Results:** Self-reported DH prevalence was 54.3% (n = 406), substantially exceeding global estimates. Among affected individuals, mean DHEQ score was 0.56 ± 0.19, indicating moderate-to-substantial quality-of-life impact. Functional limitations were most affected, particularly enjoyment of eating and drinking (0.72 ± 0.21). Significant associations were identified between higher DHEQ scores and age extremes (<18 and >35 years; *p* < 0.001), higher income levels (*p* = 0.032), fewer teeth (*p* = 0.040), and dental pain presence (*p* = 0.009). Sex, residence, education, and employment showed no significant associations. **Conclusions:** More than half of Saudi adults reported DH symptoms, representing a significant public health concern with substantial quality of life implications. Prevalence substantially exceeds global estimates, highlighting the need for targeted interventions. Age, income, tooth count, and pain presence emerged as key factors. These findings support developing population-specific prevention strategies, particularly targeting younger and older adults with tooth loss.

## 1. Introduction

Dentinal hypersensitivity (DH) is a chronic dental condition marked by transient, sharp pain arising from exposed dentin in response to thermal, tactile, evaporative, osmotic, chemical, or electrical stimuli, with no other attributable pathology [1]. This condition impairs quality of life and can complicate dental treatment. Reported DH prevalence varies dramatically, from 1.34% to 98%, reflecting differences in study populations, selection criteria, and diagnostic methods [2,3,4,5,6,7,8,9]. The global prevalence is anticipated to increase as more individuals maintain their teeth into older ages [9,10,11,12]. Higher prevalence rates have been reported in middle-aged adults, although the specific age-related factors remain unclear. Interestingly, DH prevalence appears to decline with age, possibly due to the deposition of reparative dentin and the natural obliteration of dentinal tubules [13]. Moreover, the lifestyle of young adults, such as frequent consumption of acidic foods and drinks and parafunctional habits, can promote the onset of DH [14]. Additionally, a higher prevalence of DH has been reported in females, which can be attributed to their superior oral hygiene practices and more regular dental attendance [4,15].

There are various potential causes and predisposing factors leading to DH, most commonly related to enamel loss and subsequent dentin exposure, which is naturally sensitive due to its connection to the dental pulp. Enamel loss may result from attrition, abrasion, or erosion. Erosion and abrasion are often identified as the primary contributors to tooth wear and may act additively or synergistically in causing DH [16]. Oral hygiene practices also play a role, with studies reporting associations between frequent toothbrushing, gingival recession, and DH [17]. Most studies emphasize erosion and abrasion as the main causes of DH [18,19,20]. A thorough patient history including dietary habits, oral hygiene routines, and prior dental treatments such as professional cleanings, bleaching, or restorations is essential to identify risk factors. Clinical evaluation is also key to confirming signs such as erosion, gingival recession, and exposed cervical dentin.

A variety of theories have elucidated the mechanism by which triggering stimuli provoke pain, including the odontoblastic transduction theory, neural theory, and hydrodynamic theory [21]. The latter one is the most accepted theory, which was proposed by Brannstrom and his colleagues [22,23]. This theory suggests that rapid changes in the fluid movement within the dentinal tubules, triggered by stimuli, activate sensory nerves in the pulp and inner dentin regions of the tooth. At a microscopic level, the dentinal tubules are more numerous and wider in hypersensitive dentin compared to non-sensitive dentin [2]. As a result, these features enhance fluid movement through the dentin, leading to an increase in stimulus transmission and subsequent pain response [22]. Overall, these findings support the hypothesis that dentinal pain is mediated by a hydrodynamic mechanism.

DH can profoundly interfere with quality of life by affecting daily living, like dietary habits and personal care routines. The intensity of DH pain ranges from mild discomfort to severe episodes triggered most commonly by cold stimuli [24,25], though individual teeth may respond differently to various stimuli. These fluctuations, occasionally influenced by neurogenic inflammation and chronic low-level nerve stimulation [8], can disrupt eating, drinking, and oral hygiene practices, thereby diminishing overall quality of life. Beyond physical discomfort, DH interferes with routine activities and psychosocial well-being. Studies demonstrate significant impairments in oral health related quality of life (OHRQoL), with patients reporting difficulty consuming hot or cold foods, reluctance to maintain brushing habits, and even challenges with breathing or speaking [26,27,28,29,30,31]. The variable severity and unpredictability of symptoms underline the substantial burden DH places on individuals’ day-to-day experiences. Considering that DH can prompt behavioral modifications and have an adverse impact on OHRQoL, it is imperative to investigate whether treatments for DH can improve an individual’s OHRQoL [32].

Although systematic evaluations are scarce, existing studies consistently demonstrate that DH significantly impairs OHRQoL and that desensitizing treatments can produce meaningful benefits. Two investigations using the generic Oral Health Impact Profile 14 (OHIP-14) found lower OHRQoL in individuals with DH versus controls [28,33], while another observed marked OHIP-14 score improvements after in-office treatment [34]. Machuca et al., using the DHEQ, a DH-specific OHRQoL measure, confirmed a substantial OHRQoL burden [35]. A randomized trial found that two occluding desensitizing agents, 5 percent calcium sodium phosphosilicate and 8 percent arginine with calcium carbonate, demonstrated sustained symptom relief, supporting the effectiveness of desensitizing agents for managing DH [36], and longitudinal studies similarly document enhanced OHRQoL following treatment [32,37]. These findings highlight the substantial negative impact of dentin hypersensitivity and the effectiveness of treatment in enhancing patients’ quality of life.

However, studies examining OHRQoL in patients with DH remain limited. Understanding the prevalence and associated factors of DH within the Saudi context is crucial for developing targeted interventions, particularly as Saudi Arabia’s Vision 2030 emphasizes mental health and well-being, areas significantly impacted by chronic oral pain conditions [38,39]. As Saudi Arabia strives to improve population health through its Vision 2030 initiative, addressing oral health issues like DH becomes vital. Therefore, this study aims to assess the prevalence and impact of DH on individuals’ quality of life among adults in Saudi Arabia and to identify the socio-demographic factors associated with this condition.

## 2. Materials and Methods

### 2.1. Study Design

A cross-sectional online survey was conducted between April and May 2023 among adults in Saudi Arabia. The study was designed and reported following the Strengthening the Reporting of Observational studies in Epidemiology (STROBE) guidelines for cross-sectional studies [40].

### 2.2. Ethical Considerations

The study was carried out in accordance with the Code of Ethics of the World Medical Association (Declaration of Helsinki). Ethical approval was obtained from the Institutional Review Board at King Saud University (IRB Number E-22-6689) approved on 13 March 2022. Informed consent was obtained from all participants prior to answering the questionnaire. This study followed the Checklist for Reporting Results of Internet E-Surveys (CHERRIES) guidelines [41]. A completed CHERRIES checklist is provided as Supplementary Materials.

### 2.3. Study Setting and Data Collection

The study was conducted nationally across Saudi Arabia using an online survey platform. The online survey was distributed through multiple channels to ensure broader representation. These included social media platforms (Twitter, Instagram, and WhatsApp groups), university networks and email lists, healthcare facility waiting areas (QR codes), and snowball sampling through initial participants.

### 2.4. Study Population

The study’s inclusion criteria are adults aged 18 years or older, residing in Saudi Arabia, able to read Arabic, and with internet access. Exclusion criteria included non-residency, inability to complete the online questionnaire, and cognitive impairment affecting response validity. Of the estimated 1200 individuals who accessed the survey link, 748 completed responses were obtained, yielding a response rate of 62.3%. The sample size was calculated using the standard formula for cross-sectional studies: n = (Z^2^α/2 × P × (1 − P))/d^2^, where Z^2^α/2 is the critical value of the normal distribution at α/2, P is the expected prevalence, and d is the precision [42]. Assuming a 95% confidence level, 50% expected prevalence (a conservative estimate), and 5% precision, the minimum required sample size was calculated accounting for a 15% non-response rate, and the target sample size was determined to be 443 [43].

The actual sample size obtained (n = 748) exceeded this calculated minimum, ensuring sufficient statistical power to detect the prevalence of DH with the desired precision. This larger sample size also allowed for more robust subgroup analyses [44].

### 2.5. Instrument

A self-administered, validated Arabic questionnaire was used for data collection. Permission was obtained from the original author, Dr. O.V. Boiko et al. [29]. The original English DHEQ was translated using forward-backward translation methodology. The validation process included (1) two bilingual dental professionals independently translated the questionnaire to Arabic; (2) a third bilingual expert reconciled differences; (3) back-translation to English by two independent translators; (4) comparison with original version, which showed >95% semantic equivalence; (5) pilot testing with 30 participants, which confirmed comprehensibility; and (6) calculation of Cronbach’s alpha for internal consistency (α = 0.89), indicating excellent reliability.

The questionnaire consisted of two sections. The first section gathered socio-demographic data, including sex, age, city of residence, educational level, job status, monthly income, tooth count, and pain experience. The second section comprised the Dentinal Hypersensitivity Experience Questionnaire (DHEQ), which included 15 items assessing participants’ experiences with DH across three domains: functional limitations (7 items), social limitations (2 items), emotional impact (6 items).

DHEQ responses were scored using a weighted aggregation method, with “Strongly agree” assigned a value of 1.0, “Agree” as 0.8, “Neutral” as 0.6, “Disagree” as 0.4, and “Strongly Disagree” as 0. The total DHEQ score was calculated for participants who reported experiencing DH within the past 12 months (n = 406). The final score was expressed as a percentage, with higher scores indicating a greater impact of hypersensitivity. Internal consistency of the Arabic DHEQ in this sample was assessed using Cronbach’s alpha (α = 0.89), indicating excellent reliability and confirming that the translated instrument maintained the psychometric properties of the original English version.

### 2.6. Data Analysis

The survey was administered online using Google Forms. Data were analyzed using IBM Statistics SPSS Version 29 for Mac; Version Descriptive statistics were used to summarize demographic characteristics and compare participants who experienced DH within the past 12 months (n = 406) to those who did not (n = 342).

DHEQ scores were analyzed using one-way ANOVA to examine differences across socio-demographic variables, including age, income, tooth count, and pain experience. Statistical significance was set at *p* < 0.05.

Bivariate analysis was chosen as an initial exploratory approach to identify associations between demographic factors and DHEQ scores. While multivariate analysis would provide adjusted associations, our study aimed to identify preliminary relationships to guide future research in the Saudi population. The cross-sectional design and exploratory nature of this first study in Saudi Arabia supported this analytical approach.

A complete case analysis approach was employed for missing data management. Incomplete responses (n = 52) were excluded from analysis. Participants with missing DHEQ items (>20% incomplete) were excluded from quality of life analyses.

## 3. Results

### 3.1. Description of the Study Population by Dentinal Hypersensitivity Status

A total of 748 participants were included in this study, and 406 (54.3%) reported experiencing DH within the past 12 months (Table 1). Compared to the group without DH, the group with DH had a slightly larger proportion of female participants (females with hypersensitivity 80.0% vs. without hypersensitivity 75.5%, *p* = 0.030). There was no difference in age between the two study groups, with the majority of participants aged between 18 and 35 years (participants with hypersensitivity 68.3% vs. without hypersensitivity 64.9%, *p* = 0.092). A larger proportion of study participants who reported no DH resided in Riyadh compared to participants with hypersensitivity (participants with hypersensitivity 73.2% vs. without hypersensitivity 81.0%, *p* = 0.007). In terms of educational level, a larger proportion of participants with DH completed more than high-school-level education compared to participants without DH (participants with hypersensitivity 76.5% vs. without hypersensitivity 65.0%, *p* = 0.003). A larger proportion of participants with DH reported not being employed at the time of the study compared to those without DH (participants with hypersensitivity 50.9% vs. without hypersensitivity 36.5%, *p* = 0.001). There was no significant difference in terms of income level between the two study groups (*p* = 0.22).

In terms of the number of teeth present, a slightly higher proportion of participants without DH reported having 20 teeth or more compared to the group with DH (participants with hypersensitivity 75.8% vs. without hypersensitivity 78.4%, *p* = 0.030). A significantly larger proportion of participants with DH reported experiencing pain compared to those without DH (participants with hypersensitivity 85.9% vs. without hypersensitivity 45.7%, *p* < 0.001).

### 3.2. Dentinal Hypersensitivity Experience (DHEQ) Scores for Participants with Dentinal Hypersensitivity by Subscale

After excluding participants without DH, a total of 406 participants were included in the sub-analysis to calculate DHEQ scores. The overall mean (SD) score for participants with DH was 0.56 (0.19) (Table 2).

**Functional Limitations**: The mean score for questions related to the impact of DH on functional limitations ranged from 0.72 to 0. The mean (SD) score for the statement “Having the sensations in my teeth takes a lot of the pleasure out of eating and drinking” was 0.72 (SD = 0.21). Similarly, the mean (SD) score for the statement “There have been times when I have had problems eating ice cream because of these sensations” was 0.72 (SD = 0.24). The average score for the statement “I have to change the way I eat or drink certain things” was 0.66 (SD = 0.26). The item “I have to change the way I eat or drink certain things” averaged 0.66 (SD = 0.26), while “When eating some foods, I have made sure they don’t touch certain teeth” scored 0.61 (SD = 0.25). The average score for the item “Because of the sensations, I take longer than others to finish a meal” had a mean (SD) of 0.51 (SD = 0.28). Lastly, “I have to be careful how I breathe on a cold day” had a mean (SD) score of 0.54 (SD = 0.25).

**Social Limitations:** The mean score for questions related to the impact of DH on social limitations ranged from 0.50 to 0. The mean (SD) score for “I have to be careful what I eat when I am with others because of the sensations in my teeth” was 0.50 (SD = 0.27). Similarly, “Going to the dentist is hard for me because I know it is going to be painful as a result of sensations in my teeth” had a mean (SD) score of 0.51 (SD = 0.26).

**Emotional Impact:** The emotional impact scores ranged from 0.46 to 0. The item “I’ve been anxious that something I eat or drink might cause sensations in my teeth” had a mean (SD) of 0.56 (SD = 0.27). Additionally, “The sensations in my teeth have been irritating” scored 0.48 (SD = 0.25), and “The sensations in my teeth have been annoying” scored 0.59 (SD = 0.28). Items reflecting self-perception, such as “Having these sensations in my teeth makes me feel old”, averaged 0.46 (SD = 0.26), while “makes me feel damaged” had a mean (SD) of 0.49 (SD = 0.26), and “makes me feel as though I am unhealthy” scored 0.47 (SD = 0.26).

### 3.3. DHEQ Scores by Participants’ Characteristics

Table 3 illustrates the experiences of dentinal hypersensitivity (DHEQ) based on demographic characteristics. Out of the eight demographic factors studied, four factors were significantly associated with DHEQ scores. First, age was significantly associated with DHEQ scores, with participants under 18 years scoring 0.64 (SD = 0.10), those aged 18–35 scoring 0.53 (SD = 0.19), and those over 35 scoring 0.63 (SD = 0.17) (*p* < 0). Second, monthly income level was significantly associated with DHEQ scores, with participants earning less than SR 5000 scoring 0.53 (SD = 0.19), those whose income was between SR 5000 and SR 9999 scoring 0.55 (SD = 0.21), those earning more than SR 10,000 scoring 0.58 (SD = 0.16), and those who did not disclose scoring 0.61 (SD = 0.20) (*p* = 0). Third, tooth count was also associated with DHEQ scores, with participants having 1–9 teeth scoring 0.64 (SD = 0.15), those with 10–19 teeth scoring 0.52 (SD = 0.20), and those with more than 20 teeth scoring 0.56 (SD = 0.19) (*p* = 0). Fourth, pain presence significantly impacted scores as well, with participants experiencing pain scoring 0.57 (SD = 0.20) compared to 0.51 (SD = 0.16) for those without pain (*p* = 0.009).

Sex was not associated with DHEQ scores, with males scoring 0.56 (SD = 0.20) and females 0.56 (SD = 0.19) (*p* = 0). City of residence showed no significant impact, with scores of 0.55 (SD = 0.19) for Riyadh residents and 0.58 (SD = 0.19) for others (*p* = 0). Educational level was also not significantly associated, with scores ranging from 0.54 to 0.60 across different education levels (*p* = 0). Employment status showed no significant association either, with scores of 0.55 (SD = 0.20) for non-employed participants, 0.56 (SD = 0.20) for full-time, 0.55 (SD = 0.17) for part-time, and 0.59 (SD = 0.09) for retirees (*p* = 0.78).

## 4. Discussion

To our knowledge, this is the first study assessing the prevalence of DH among Saudi adults and evaluating its impact on quality of life. Three main findings emerged from the data. First, more than half of the study participants reported having symptoms of DH within the past year. Second, participants with DH reported that this condition interfered with their quality of life. Third, several participant-related factors were associated with DH.

### 4.1. Prevalence of Dentinal Hypersensitivity in Saudi Arabia

More than half of the surveyed population reported experiencing DH, a prevalence that falls within the upper range of global estimates, which vary between 3% and 57% [3,10]. This suggests that DH may represent a more pronounced oral health concern in Saudi Arabia compared to other regions. Despite its evident impact, the prevalence and epidemiology of DH remain relatively understudied, particularly within the Saudi population. The limited number of region-specific studies highlights a significant gap in the literature and underscores the need for further research to better understand the distribution, risk factors, and management of this condition.

### 4.2. Impact on Quality of Life and Functional Limitations

The findings also underscore the direct impact of DH on individuals’ quality of life, particularly the discomfort experienced during routine activities such as eating and drinking, as evidenced by responses on the Dentine Hypersensitivity Experience Questionnaire (DHEQ). This aligns with Bekes et al. (2009), who found that DH significantly impairs oral health-related quality of life [28]. Quality of life encompasses an individual’s overall well-being, including physical, mental, and social dimensions as well as autonomy and functional ability [45]. In Saudi Arabia, enhancing quality of life is a central goal of the Saudi Vision 2030 initiative [38], which emphasizes healthier lifestyles, improved preventive healthcare services, and greater public awareness. Poor oral health is increasingly recognized as a key determinant of diminished quality of life, and recent evidence points to DH as a contributing factor [28,33,35,36]. The condition causes tangible discomfort during common daily activities such as eating, drinking, and brushing, thereby compromising both comfort and oral function [1].

### 4.3. Sociodemographic Factors Associated with DH

Several participant-related factors were significantly associated with DH. Age, income, and tooth count influenced both the presence and impact of the condition. Individuals younger than 18 years and those older than 35 years reported higher DHEQ scores, indicating a greater impact of sensitivity on their quality of life. This partially aligns with previous research, which has shown that DH is most reported among middle-aged adults [28]. However, the specific age-related factors contributing to this condition remain unclear. Potential explanations could include age-related changes in enamel wear, gingival recession, or variations in oral hygiene practices across different life stages. Income also emerged as a significant factor, with participants in higher income brackets reporting elevated DHEQ scores. This may reflect increased health awareness and a lower threshold for perceiving or reporting discomfort among individuals from higher socioeconomic backgrounds. These findings are consistent with previous research by Locker (2000), which suggested that individuals with higher socioeconomic status are more likely to report oral health concerns and actively seek care [46]. Additionally, a notable correlation was observed between tooth loss and higher DHEQ scores, potentially indicating that individuals with fewer teeth may experience more severe underlying dental conditions. This supports findings by Nuttall et al. (2001), who reported an association between tooth loss and increased oral health problems, including DH [47].

### 4.4. Methodological Considerations and Bias Assessment

The observed prevalence of 54.3% substantially exceeds global estimates, necessitating careful consideration of potential biases. Several factors may contribute to this elevated prevalence. First, self-selection bias inherent in online survey methodology may have attracted participants experiencing symptoms, thereby inflating prevalence estimates compared to population-based clinical examinations. Second, our sample was predominantly female (80%), urban (73% from Riyadh), and highly educated (65% >high school), which may not represent the broader Saudi population. According to national statistics, Saudi Arabia has more balanced demographics (51% male, 49% female), with approximately 65% urban residence [48]. Third, the self-reported nature of DH without clinical confirmation may introduce measurement bias, though validated questionnaires like DHEQ have shown good correlation with clinical diagnosis.

Findings should therefore be interpreted as estimates for educated, urban Saudi adults with internet access rather than the general Saudi population. The convenience sampling methodology and online survey format may have systematically excluded rural populations, older adults with limited digital literacy, and individuals with lower educational attainment. Future studies should employ population-based sampling strategies, including rural communities and diverse educational backgrounds, to improve generalizability and provide more representative prevalence estimates for the entire Saudi population.

### 4.5. Mental Health and Saudi Vision 2030 Implications

The psychological impact of DH, particularly anxiety related to eating and drinking, aligns with Saudi Vision 2030’s emphasis on mental health and well-being [38,39]. Recent research by Zimmer et al. (2025) demonstrated a significant association between DH and anxiety in young adults, supporting our findings of elevated anxiety scores (DHEQ anxiety items: 0.46–0.59) [49]. This evidence indicates that DH management should incorporate psychological support alongside clinical treatment.

### 4.6. Public Health Implications and Clinical Significance

From a public health perspective, the high prevalence of DH observed in this study highlights the need to integrate targeted care for this condition into national dental health programs, in alignment with the goals of Saudi Vision 2030 [38]. Implementing early intervention strategies, public awareness campaigns, and routine screening initiatives could play a crucial role in reducing the burden of this condition and enhancing overall oral health outcomes. Similar approaches have proven effective in other countries, such as the United Kingdom, where targeted oral health programs have led to measurable improvements in population-level oral health indicators [50]. Additionally, individuals with fewer teeth, who in this study also reported higher sensitivity, may benefit from specialized dental care and rehabilitation services aimed at managing more advanced oral health issues and improving quality of life.

### 4.7. Recommendations for Dental Practice

Based on our findings, we recommend: (1) implementation of routine DH screening in clinical practice using validated questionnaires; (2) development of Arabic patient education materials addressing DH management; (3) training programs for dental professionals in DH diagnosis and treatment protocols; (4) establishment of referral pathways for complex cases requiring specialized intervention; and (5) integration of quality of life assessment in DH treatment planning.

### 4.8. Recommendations for Future Research

Future studies should (1) conduct longitudinal investigations to establish causal relationships between identified factors and DH development; (2) perform clinical validation studies using standardized diagnostic criteria alongside self-reported measures; (3) include rural populations and diverse educational backgrounds to improve generalizability; (4) investigate effectiveness of culturally-adapted interventions for the Saudi population; and (5) conduct multivariate analyses to identify independent predictors of DH impact on quality of life.

### 4.9. Study Limitations

This study has several limitations that should be acknowledged. First, the reliance on self-reported DH without clinical confirmation may introduce measurement bias, though validated questionnaires like DHEQ have shown good correlation with clinical diagnosis. Second, self-selection bias inherent in online surveys may have systematically attracted symptomatic participants, potentially inflating prevalence estimates. Third, our sample was not representative of the general Saudi population, being predominantly female, urban, and highly educated, which limits generalizability to rural and less educated populations. Fourth, the cross-sectional design prevents establishment of causal relationships between identified factors and DH presence or severity. Fifth, the absence of multivariate analysis limits our ability to identify independent predictors while controlling for confounding variables. Sixth, recall bias may affect the accuracy of self-reported symptoms and experiences.

## 5. Conclusions

In conclusion, more than half of Saudi adults in this study reported symptoms of DH, placing the observed prevalence within the upper range of global estimates and highlighting a significant public health concern. Key factors associated with increased sensitivity included age, income level, and tooth count, emphasizing the importance of addressing this condition through targeted prevention and management strategies. These findings support the need for tailored oral health interventions, particularly for younger and older adults as well as individuals with fewer teeth, to reduce the burden of DH. This study provides insights that can guide public health initiatives and clinical practices in alignment with the goals of Saudi Vision 2030 to enhance population health and overall quality of life. Implementing early interventions, public awareness campaigns, and routine screening programs may help mitigate the impact of this condition and promote better oral health across the population. Future longitudinal studies are recommended to establish causal relationships and to assess the effectiveness of interventions aimed at managing DH over time.

## Figures and Tables

**Table 1:** Demographic characteristics of the sample according to self-reported dentinal hypersensitivity experience within the previous 12 months (n = 748).

		Dentinal Hypersensitivity Experience		
		Yes Frequency (%) n = 406	No Frequency (%) n = 342	*p*-Value *
Sex	Male Female	81 (20) 325 (80)	93 (26.7) 255 (75.5)	**0.030**
Age	Less than 18 years Between 18 and 35 years More than 35 years	3 (0.8) 271 (68.3) 117 (29.9)	9 (2.6) 222 (64.9) 111 (32.5)	0.092
City	Riyadh Others	297 (73.2) 109 (26.8)	282 (81) 66 (19)	**0.007**
Educational level	Less than high school degree High school degree More than high school degree	18 (4.4) 124 (30.5) 264 (65)	9 (2.6) 72 (20.9) 264 (76.5)	**0.003**
Job status	Not employed Full time Part time Retired	148 (36.5) 193 (47.5) 32 (7.9) 33 (8.1)	177 (50.9) 135 (38.8) 15 (4.3) 21 (6)	**0.001**
Monthly income (SR)	Less than 5000 Between 5000 and 9999 More than 10,000 Don’t want to declare	144 (35.5) 78 (19.2) 105 (25.9) 79 (19.5)	129 (37.1) 51 (14.7) 84 (24.1) 84 (24.1)	0.218
Tooth count	1–9 teeth 10–19 teeth More than 20	34 (10.9) 54 (13.5) 273 (75.8)	36 (6) 54 (15.5) 303 (78.4)	**0.030**
Pain	Yes No	349 (85.9) 57 (14)	159 (45.7) 189 (54.3)	**<0.001**

* Chi-square was used for categorical variables, and *t*-test was used for continuous variables; *p*-value < 0.05 is bolded.

**Table 2:** Average mean score of dentinal hypersensitivity experiences (DHEQ) among participants reporting experiencing dentinal hypersensitivity in the past 12 months (n = 406).

DHEQ	Mean (SD)
Having the sensations in my teeth takes a lot of the pleasure out of eating and drinking	0.72 (0.21)
It takes a long time to finish some foods and drinks because of these sensations in my teeth	0.60 (0.19)
There have been times when I have had problems eating ice cream because of these sensations	0.72 (0.24)
I have to change the way I eat or drink certain things	0.66 (0.26)
I have to be careful how I breathe on a cold day	0.54 (0.25)
When eating some foods, I have made sure they don’t touch certain teeth	0.61 (0.25)
Because of the sensations, I take longer than others to finish a meal	0.51 (0.28)
I have to be careful what I eat when I am with others because of the sensations in my teeth	0.50 (0.27)
Going to the dentist is hard for me because I know it is going to be painful as a result of sensations in my teeth	0.51 (0.26)
I’ve been anxious that something I eat or drink might cause sensations in my teeth	0.56 (0.27)
The sensations in my teeth have been irritating	0.48 (0.25)
The sensations in my teeth have been annoying	0.59 (0.28)
Having these sensations in my teeth makes me feel old	0.46 (0.26)
Having these sensations in my teeth makes me feel damaged	0.49 (0.26)
Having these sensations in my teeth makes me feel as though I am unhealthy	0.47 (0.26)
Total	0.56 (0.19)

**Table 3:** Dentinal hypersensitivity experiences (DHEQ) by participants’ characteristics (n = 406).

		DHEQ Score	
		Mean (SD) n = 406	*p*-Value *
Sex	Male Female	0.56 (0.20) 0.56 (0.19)	0.935
Age	Less than 18 years Between 18 and 35 years More than 35 years	0.64 (0.10) 0.53 (0.19) 0.63 (0.17)	**<0.001**
City	Riyadh Others	0.55 (0.19) 0.58 (019)	0.216
Educational level	Less than high school degree High school degree More than high school degree	0.60 (0.16) 0.54 (0.21) 0.57 (0.19)	0.366
Job status	Not employed Full-time Part-time Retired	0.55 (0.20) 0.56 (0.20) 0.55 (0.17) 0.59 (0.09)	0.783
Monthly income (SR)	Less than 5000 Between 5000 and 9999 More than 10,000 Don’t want to declare	0.53 (0.19) 0.55 (0.21) 0.58 (0.16) 0.61 (0.20)	**0.032**
Tooth count	1–9 teeth 10–19 teeth More than 20	0.64 (0.15) 0.52 (0.20) 0.56 (0.19)	**0.040**
Pain	Yes No	0.57 (0.20) 0.51 (0.16)	**0.009**

* One-way ANOVA. *p*-value < 0.05 is bolded.

## Data Availability

The relevant data are contained within the article.

## Supplementary Materials

The following supporting information can be downloaded at: https://www.mdpi.com/article/10.3390/dj13080353/s1, File S1: CHERRIES checklist.

## Abbreviations

The following abbreviations are used in this manuscript:

DH	Dentinal hypersensitivity
DHEQ	Dentinal Hypersensitivity Experience Questionnaire
OHRQoL	Oral-health-related quality of life
STROBE	STrengthening the Reporting of OBservational studies in Epidemiology
IRB	Institutional Review Board
