# Supplementary material for: The Prevalence and Impact of Dentinal Hypersensitivity on Adults’ Quality of Life in Saudi Arabia
Source: Dent J (Basel). 2025 Aug 4;13(8):353. doi: 10.3390/dj13080353 (PMC12384756; doi:10.3390/dj13080353)
Supplement: Supplementary file 1 [file dentistry-13-00353-s001.zip › dentistry-3746577-supplementary.pdf]

Checklist for Reporting Results of Internet E-Surveys (CHERRIES)

Checklist Item	***Explanation***	***Page Number***
Describe survey design	Describe target population, sample frame. Is the sample a convenience sample? (In "open" surveys this is most likely.)	Page 4-5
Cross-sectional online survey conducted between April and May 2023 among adults in Saudi Arabia. Target population: adults aged ≥18 years residing in Saudi Arabia. Convenience sample using multiple recruitment channels.		
IRB approval	Mention whether the study has been approved by an IRB.	Page 4
Ethical approval obtained from the Institutional Review Board at King Saud University (IRB Number E-22-6689).		
Informed consent	Describe the informed consent process. Where were the participants told the length of time of the survey, which data were stored and where and for how long, who the investigator was, and the purpose of the study?	Page 4
Digital informed consent obtained from all participants prior to questionnaire access. Participants informed about 10-15 minute duration, data storage for research purposes, investigator contact information, and study objectives.		
Data protection	If any personal information was collected or stored, describe what mechanisms were used to protect unauthorized access.	Page 5
No personally identifiable information collected. Data stored securely on password-protected university systems. IP addresses used only for duplicate detection.		
Development and testing	State how the survey was developed, including whether the usability and technical functionality of the electronic questionnaire had been tested before fielding the questionnaire.	Page 5
Validated Arabic DHEQ using forward-backward translation. Pilot testing with 30 participants. Cronbach's alpha = 0.89. Technical functionality tested across multiple browsers and devices.		
Open survey versus closed survey	An "open survey" is a survey open for each visitor of a site, while a closed survey is only open to a sample which the investigator knows (password-protected survey).	Page 4-5
Open survey accessible via public links distributed through multiple channels including social media, university networks, and healthcare facilities.		
Contact mode	Indicate whether or not the initial contact with the potential participants was made on the Internet. (Investigators may also send out questionnaires by mail and allow for Web-based data entry.)	Page 4-5
Initial contact made through Internet-based channels: social media platforms, email lists, QR codes, and online networks.		
Advertising the survey	How/where was the survey announced or advertised? Some examples are offline media (newspapers), or online (mailing lists – If yes, which ones?) or banner ads (Where were these banner ads posted and what did they look like?). It is important to know the wording of the announcement as it will heavily influence who chooses to participate. Ideally the survey announcement should be published as an appendix.	Page 4-5
Survey distributed through: social media platforms (Twitter, Instagram, WhatsApp groups), university networks and email lists, healthcare facility waiting areas (QR codes), snowball sampling.		
Web/E-mail	State the type of e-survey (eg, one posted on a Web site, or one sent out through e-mail). If it is an e-mail survey, were the responses entered manually into a database, or was there an automatic method for capturing responses?	Page 5
Web-based survey posted on Google Forms platform with automatic response capture and database entry.		
Context	Describe the Web site (for mailing list/newsgroup) in which the survey was posted. What is the Web site about, who is visiting it, what are visitors normally looking for? Discuss to what degree the content of the Web site could pre-select the sample or influence the results. For example, a survey about vaccination on a anti-immunization Web site will have different results from a Web survey conducted on a government Web site	Page 4-5

Multiple platforms used to minimize selection bias: academic networks, healthcare settings, general social media. No single platform that would pre-select for specific attitudes toward dental health.		
Mandatory/voluntary	Was it a mandatory survey to be filled in by every visitor who wanted to enter the Web site, or was it a voluntary survey?	Page 4
Voluntary participation. No mandatory requirements for website access or other services.		
Incentives	Were any incentives offered (eg, monetary, prizes, or non-monetary incentives such as an offer to provide the survey results)?	Not mentioned
No incentives offered for participation.		
Time/Date	In what timeframe were the data collected?	Page 4
Data collected between April and May 2023.		
Randomization of items or questionnaires	To prevent biases items can be randomized or alternated.	Not applicable
No randomization of items. Standardized validated questionnaire (DHEQ) used with fixed item order.		
Adaptive questioning	Use adaptive questioning (certain items, or only conditionally displayed based on responses to other items) to reduce number and complexity of the questions.	Page 5
Adaptive questioning used: participants reporting no DH symptoms in past 12 months were excluded from DHEQ scoring but included in prevalence calculations.		
Number of Items	What was the number of questionnaire items per page? The number of items is an important factor for the completion rate.	Page 5
Total 23 questions: 8 demographic questions + 15 DHEQ items. Multiple items per page for logical grouping.		
Number of screens (pages)	Over how many pages was the questionnaire distributed? The number of items is an important factor for the completion rate.	Page 5
Multi-page format: informed consent page, demographic section, DH screening, DHEQ section (for eligible participants).		
Completeness check	It is technically possible to do consistency or completeness checks before the questionnaire is submitted. Was this done, and if "yes", how (usually JAVAScript)? An alternative is to check for completeness after the questionnaire has been submitted (and highlight mandatory items). If this has been done, it should be reported. All items should provide a non-response option such as "not applicable" or "rather not say", and selection of one response option should be enforced.	Page 5
All questions mandatory with built-in validation. Participants could not submit incomplete forms. Progress indicator showed completion status.		
Review step	State whether respondents were able to review and change their answers (eg, through a Back button or a Review step which displays a summary of the responses and asks the respondents if they are correct).	Not mentioned
Standard Google Forms functionality allowed participants to review and modify responses before final submission.		
Unique site visitor	If you provide view rates or participation rates, you need to define how you determined a unique visitor. There are different techniques available, based on IP addresses or cookies or both.	Page 5
IP address tracking through Google Forms to identify unique visitors and prevent duplicate submissions.		
View rate (Ratio of unique survey visitors/unique site visitors)	Requires counting unique visitors to the first page of the survey, divided by the number of unique site visitors (not page views!). It is not unusual to have view rates of less than 0.1 % if the survey is voluntary.	Not calculable
Cannot be calculated due to multiple distribution channels and lack of unified site visitor tracking.		

Participation rate (Ratio of unique visitors who agreed to participate/unique first survey page visitors)	Count the unique number of people who filled in the first survey page (or agreed to participate, for example by checking a checkbox), divided by visitors who visit the first page of the survey (or the informed consents page, if present). This can also be called "recruitment" rate.	Not calculable
Cannot be precisely calculated due to multiple distribution channels and snowball sampling methodology.		
Completion rate (Ratio of users who finished the survey/users who agreed to participate)	The number of people submitting the last questionnaire page, divided by the number of people who agreed to participate (or submitted the first survey page). This is only relevant if there is a separate "informed consent" page or if the survey goes over several pages. This is a measure for attrition. Note that "completion" can involve leaving questionnaire items blank. This is not a measure for how completely questionnaires were filled in. (If you need a measure for this, use the word "completeness rate".)	Page 6
100% completion rate - only completed responses (n=748) included in analysis. Incomplete responses automatically excluded by Google Forms settings.		
Cookies used	Indicate whether cookies were used to assign a unique user identifier to each client computer. If so, mention the page on which the cookie was set and read, and how long the cookie was valid. Were duplicate entries avoided by preventing users access to the survey twice; or were duplicate database entries having the same user ID eliminated before analysis? In the latter case, which entries were kept for analysis (eg, the first entry or the most recent)?	Not applicable
Google Forms standard cookies for session management. No custom cookies for user tracking implemented.		
IP check	Indicate whether the IP address of the client computer was used to identify potential duplicate entries from the same user. If so, mention the period of time for which no two entries from the same IP address were allowed (eg, 24 hours). Were duplicate entries avoided by preventing users with the same IP address access to the survey twice; or were duplicate database entries having the same IP address within a given period of time eliminated before analysis? If the latter, which entries were kept for analysis (eg, the first entry or the most recent)?	Page 5
IP address tracking enabled to prevent multiple submissions from same user. No time restriction specified.		
Log file analysis	Indicate whether other techniques to analyze the log file for identification of multiple entries were used. If so, please describe.	Not applicable
Standard Google Forms duplicate prevention mechanisms used. No additional log file analysis conducted.		
Registration	In "closed" (non-open) surveys, users need to login first and it is easier to prevent duplicate entries from the same user. Describe how this was done. For example, was the survey never displayed a second time once the user had filled it in, or was the username stored together with the survey results and later eliminated? If the latter, which entries were kept for analysis (eg, the first entry or the most recent)?	Not applicable
Open survey design. Duplicate prevention through IP address tracking only.		
Handling of incomplete questionnaires	Were only completed questionnaires analyzed? Were questionnaires which terminated early (where, for example, users did not go through all questionnaire pages) also analyzed?	Page 6
Only completed questionnaires analyzed (n=748). Incomplete responses automatically excluded.		
Questionnaires submitted with an atypical timestamp	Some investigators may measure the time people needed to fill in a questionnaire and exclude questionnaires that were submitted too soon. Specify the timeframe that was used as a cut-off point, and describe how this point was determined.	Not applicable
No time-based exclusion criteria applied. All submitted responses within collection period included.		

Statistical correction	Indicate whether any methods such as weighting of items or propensity scores have been used to adjust for the non-representative sample; if so, please describe the methods.	Page 6
No statistical weighting or propensity score adjustments applied. Limitations of convenience sampling acknowledged in discussion.		

This checklist has been modified from Eysenbach G. Improving the quality of Web surveys: the Checklist for Reporting Results of Internet E-Surveys (CHERRIES). J Med Internet Res. 2004 Sep 29;6(3):e34 [erratum in J Med Internet Res. 2012; 14(1): e8.]. Article available at https://www.jmir.org/2004/3/e34/; erratum available https://www.jmir.org/2012/1/e8/. Copyright ©Gunther Eysenbach. Originally published in the Journal of Medical Internet Research, 29.9.2004 and 04.01.2012.

This is an open-access article distributed under the terms of the Creative Commons Attribution License (https://creativecommons.org/licenses/by/2.0/), which permits unrestricted use, distribution, and reproduction in any medium, provided the original work, first published in the Journal of Medical Internet Research, is properly cited.
